# Editorial: Modulation of antibody-mediated effector functions in natural killer cells: protective and detrimental effects in infectious diseases

**DOI:** 10.3389/fimmu.2024.1407889

**Published:** 2024-04-30

**Authors:** Ricciarda Galandrini, Sachiyo Yoshio, Cristina Capuano

**Affiliations:** ^1^ Department of Experimental Medicine, Sapienza University of Rome, Rome, Italy; ^2^ Department of Liver Diseases, National Center for Global Health and Medicine, Ichikawa, Japan; ^3^ Departmental Faculty of Medicine and Surgery, UniCamillus-Saint Camillus International University of Health and Medical Sciences, Rome, Italy

**Keywords:** NK cells, ADCC, FCγRIIIA/CD16, viral infections, Fc-mediated antibody functions

This Research Topic collects scientific contributions focusing on factors affecting the amplitude and the functional outcome of Fc-FcγRIIIA interaction in human NK cells, in the context of viral infections.

NK cells are main effectors of Fc-dependent antibody (Ab) functions through the expression of FcγRIIIA/CD16, a type I, low affinity, activating receptor, recognizing IgG-opsonized cells or IgG-containing immune complexes. CD16 engagement activates the full spectrum of NK cell effector functions against viruses by promoting the killing of infected cells, as well as the production of cytokines and chemokines, through which they interface with adaptive immune responses and participate to pro-inflammatory tissue damage.

In this context, review from Vanderven and Kent focuses on the balance between protective and detrimental pro-inflammatory outcomes, by analyzing data form passive immunotherapy trials in severe respiratory viral infections including influenza, COVID-19, and respiratory syncytial virus disease. Overall reported data indicate that, at early stages of infection, the treatment with convalescent plasma or hyperimmune intravenous immunoglobulin may exert a protective role, based on Ab-mediated neutralizing and Fc functions. Differently, in advanced stage, the same treatment may contribute to inflammation and immunopathology basically through Fc-mediated Ab functions, outweighing any beneficial effects from viral clearance. The protective and harmful effects of Fc-mediated functions is discussed in the context of NK cell-mediated Ab-dependent cellular cytotoxicity (ADCC), as possible contributor in the equilibrium between protection and pathology.

The prophylactic setting is assessed by three original reports focusing on COVID-19 vaccination. Motsoeneng et al. investigate Fc functions of anti-spike Abs induced by mRNA ChAdOx1 vaccination or natural infection with D614G or Beta Sars-CoV-2 variants, in HIV-infected patients. The study shows that although vaccinated patients developed delayed and impaired neutralizing and Ab-dependent cellular phagocytosis (ADCP) responses, they rapidly developed robust ADCC, lasting 6 months post-vaccination. In this context, ADCC is proposed as a mechanism that ensures a durable and broad vaccine-induced immunity in HIV-infected individuals. Following natural infection both ADCP and neutralizing responses were delayed in patients, with respect to healthy donors; moreover, depending on the infecting variant, minor differences in Fc-mediated responses and kinetics were observed. Notably, HIV-infected patients in ART treatment were capable to elicit a robust Fc-mediated response, of the same magnitude as the healthy controls.

The theme of immune escape and cross-protection between virus variants is addressed by Balinsky et al. The study characterizes Ab responses specific for S1, RBD, NTD, and S2 domains of the SARS-CoV-2 ancestral strain and Omicron BA.1 variant in mRNA-1273 vaccinated subjects; Ab subclasses, neutralization activity, and FcR-dependent functions were analyzed. Notably, Ab specific for RBD, NTD, and S1 were all prone to omicron BA.1 escape; on the contrary, S2 antibodies showed potent ADCC and resisted BA.1 escape, suggesting that S2 domain mediates cross-protection against Omicron BA.1 through Fc-mediated NK cell activation.

Post-vaccinal ADCC responses have been deeply characterized by Capuano et al. The study describes the impact of heterologous (adenoviral prime followed by mRNA boost) SARS-CoV-2 vaccination on FcγRIIIA/CD16 dynamics evidencing a persistent receptor downregulation correlating with the impairment of ADCC and IFNγ production in CD56dim NK cells. At variance, CD16 functional responsiveness of NKG2C+ subset, which displays amplified size and functionality in HCMV+ individuals representing the “memory” NK cell population, resulted intrinsically insensitive to CD16 levels.

The relationship between FcγRIII expression and ADCC magnitude, both in humans and in Rhesus macaque, is also addressed by Tuyishime et al. The study identifies a combination of Fc-FcR parameters in NK cells, including ADCC magnitude, FcγRIII cell-surface expression, and frequency of phosphorylated CD3ζ, contributing to inter-species differences in ADCC. They also show that FcγRIII F158V polymorphism, affecting Fc-FcR affinity, does not explain by itself inter-species differences in Fc-dependent NK effector functions. In this regard, the report from Capuano et al. shows that, despite a comparable CD16 downregulation, low affinity individuals resulted protected from post-vaccinal functional impairment, with respect to intermediate and high affinity ones.

In this context, a comprehensive analysis of FcγR polymorphisms was performed by Conley et al. in a large number of Indian-origin Rhesus macaque, Macaca mulatta. Through long-read RNA sequencing, authors describe single nucleotide polymorphisms, insertions, deletions, frame-shift mutations, and isoforms occurring at FcγR gene. The study has profound translational implications and adds new insights on Rhesus macaque type I FcγR genetic variations that may influence antibody-mediated outcomes.

Ab glycosylation is a well-recognized post-translational modification with remarkable influence on IgG effector functions. Specifically, the removal of the core fucose greatly increases the affinity for CD16, resulting in enhanced functional outcome; indeed, afucosylated mAbs are increasingly exploited to optimize the effectiveness of immunotherapies in infectious and cancer.

Report from Capuano et al. evidenced that CD16 high affinity ligation, by means of afucosylated mAb, overcame vaccine-induced and genotype-dependent functional defects, independently from post-vaccinal CD16 expression levels.

In this regard study from Karampatzakis et al. a addresses the mechanisms by which mAb afucosylation affects the dynamics of NK-opsonized target cell interactions, CD16 expression, and effector functions. Using live-cell time-lapse microscopy, authors evidence that afucosylated mAb promotes an amplified and faster killing of opsonized target cells, with respect to control mAb. Data also demonstrate a rapid shedding of CD16 from NK cell surface leading to an accelerated disassembly of the immune synapse, enabling a greater proportion of NK cells to engage multiple targets. The enhanced capability to mediate the serial killing of afucosylated mAb-opsonized targets adds new insights on the functional impact of glycoengineered mAb on NK cell lytic potential and the consequent improved effectiveness of afucosyated mAb-based immunotherapies.

## Author contributions

RG: Conceptualization, Writing – review & editing. SY: Writing – review & editing. CC: Writing – review & editing.

